# Deconstructing the intra-tumor subclonal heterogeneity of lung synchronous ground-glass nodules using whole-genome sequencing

**DOI:** 10.1038/s41392-022-00982-4

**Published:** 2022-05-27

**Authors:** Yijiu Ren, Minfang Song, Yunlang She, Huikang Xie, Hui Zheng, Chang Chen, Yiwen Zhang

**Affiliations:** 1 Department of Thoracic Surgery, Shanghai Pulmonary Hospital, Tongji University School of Medicine, Shanghai, China; 2 School of Life Science and Technology, ShanghaiTech University, Shanghai, China; 3 State Key Laboratory of Biotherapy, West China Hospital, Sichuan University and Collaborative Innovation Center for Biotherapy, Chengdu, China

**Keywords:** Cancer genomics, Cancer models

**Dear Editor,**

The persistent presence of ground-glass opacity nodules (GGN) on thin-section computed tomography (CT) usually suggests the presence of lung adenocarcinoma,^1^ which include atypical adenomatous hyperplasia (AAH), adenocarcinoma in situ (AIS), minimally invasive adenocarcinoma, and invasive adenocarcinoma (AD).^2^ Recent data indicate that up to 20% of GGN patients (3% of the screening population) are diagnosed with synchronous multiple ground-glass nodules (SM-GGNs).^3^ At present, neither auxiliary tests that could assist in differential diagnosis nor recommended strategy for the identification and treatment of GGNs exist in the clinical practice guidelines for lung cancer, although clinical evidence suggests that SM-GGNs are more complicated, making treatment decisions difficult.^1^ Recent studies using next generation sequencing suggest differences in the mutational landscape not only among inter-tumors but also intra-tumor.

In this study, we first performed whole-genome sequencing (WGS) on 15 samples from five patients with each having two GGNs and one normal control (Fig. 1a, Supplementary Fig. S1). Detailed clinical features are summarized in Supplementary Table S1. All patients were non-smoking and disease free within the 24–30 months after surgery. We first explored the somatic copy number variations (CNV) patterns using deep WGS. We found 2184 somatic CNVs (26 deletions and 2158 amplifications) in the early stages of lung AD and 2280 CNVs (72 deletions and 2208 amplifications) in AAH. The CNV heatmap and correlation analyses revealed two CNV patterns. The independent subtype was characterized by independent lineage relationships (patients 1 and 3). Primary lung cancers and their matched AAHs shared considerably fewer global CNV patterns (Fig. 1b). The CNVs across the genomes between primary lung AD and AAH were found to correlate less with each other in two patients (Fig. 1b). However, the parallel subtype was characterized by parallel lineage relationships (patients 2, 4, 5). As indicated by the CNV heatmap, primary lung ADs and their matched AAHs generated similar global CNV patterns (Fig. 1c). The CNVs across the genomes between primary lung ADs and AAHs correlated positively with each other, with a correlation coefficient >0.8 for all three patients (Fig. 1c). the amplifications or deletions did not share the patterns among different patients. Interestingly, the histologic characteristics of the two lesions of each patient of parallel lineage relationships (patients 2, 4, 5) showed similarities (Fig. 1a). In patient 2, both AIS and AAH showed mild atypical pneumocytes proliferation while only AIS showed thickened alveolar walls. For patients 4 and 5, thickened alveolar walls and more-marked atypical proliferating pneumocytes could be observed in both primary lung AD and AAH, while AAH was diagnosed because of the small size of lesion. The histologic characteristics of the two lesions of each patient of independent lineage relationships (patients 1, 3) showed differences. In patient 1, AAH did not show thickened alveolar walls or more-marked atypical pneumocytes proliferation, whereas AIS showed both characteristics. In patient 3, AAH showed only thickened alveolar walls, whereas primary lung AD showed both characteristics (Fig. 1a).

**Fig. 1:**
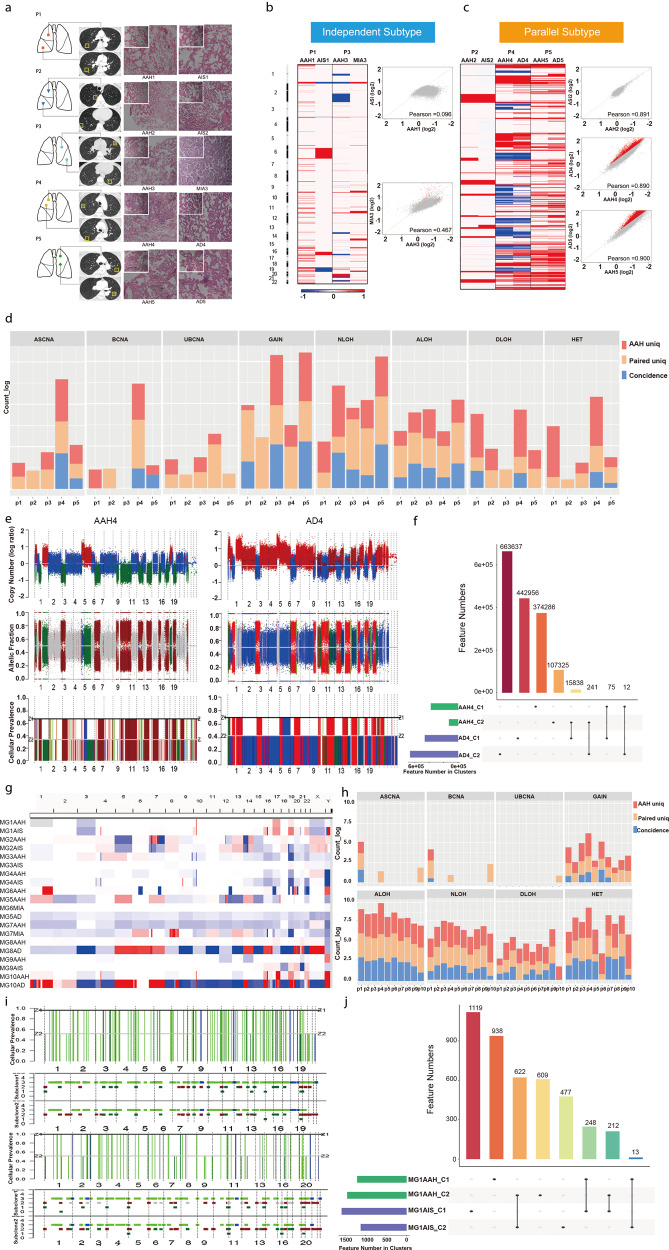
The molecular profiling identify intra-tumor subclonal heterogeneity multiple ground-glass nodules for the whole-genome sequencing. **a** Schematic of the five synchronous multiple ground-glass nodules for the whole-genome sequencing. The left side shows the location of all lesions in the lung models, with colorful dots representing the lesions. The middle side shows the CT scans of all lesions, with the yellow block chart showing the exact locations of the lesions. The right side shows the pathology sections of all lesions. P represents each patient. **b**, **c** Heatmap and plot of the somatic copy number variations of the five synchronous multiple ground-glass opacity lesions for the whole-genome sequencing. In the heatmap, the horizontal coordinates represent lesions, whereas the vertical coordinates represent chromosomes, with the red bar representing copy number gain and the blue bar representing copy number loss. The Pearson correlation coefficients were calculated. **d** Deconstructing CNV and LOH types. Various types of copy number and LOH were inferred using Titan program. The CNV and LOH features were extracted from result files following the iterations of cellular subclonal cluster analysis that yielded the best clonal structure state. The same loci with the same types of features were compared for commonalities or uniquenesses across the paired SM-GGN samples. Allele-specific copy number amplification (ASCNA), Balanced copy number amplification (BCNA), Unbalanced copy number amplification (UBCNA), One-copy gain (GAIN), copy neutral LOH (NLOH), Amplification LOH (ALOH), Hemizygous deletion LOH (DLOH), heterozygous (HET). **e** Clonal structure of AAH4 (left) and AD4 (right) of patient 4. Top panel: Copy number is represented as the log ratio of tumor and normal read depth. Discrete copy number status shown is predicted as either a hemizygous deletion (HEMD; green), copy neutral (NEUT; blue), or gain/amplification (AMP; red). Middle panel: Allelic ratios are computed as the proportion of reads matching the reference genome. The LOH status shown is HET (gray), LOH (green), NLOH (blue), or ASCNA (red). Lower panel: the sample cellular prevalence estimates (proportion of sample) for a subclonal cluster ‘Z1’ and ‘Z2’. **f** CNV/LOH features shared by subclones. **g** Somatic CNVs were detected using HHMcopy program for the low-depth WGS data, and the CNV segments are displayed as amplified CNV (red) and deletion CNV (blue) among chromosomes of all 10 GGN samples of 10 patients. **h** Various types of copy number and LOH were inferred by Titan program. The CNV and LOH features were extracted from result files following the iterations of cellular subclonal cluster analysis that yielded the best clonal structure state. The same loci with the same types of features were compared for commonalities or uniquenesses across the paired SM-GGN samples. **i** Clonal structure of MG1AAH (top) and MG1AIS (bottom) of patient MG1. Top panel: The sample cellular prevalence estimates (proportion of sample) for a subclonal cluster ‘Z1’ and ‘Z2’. Lower panel: CNV/LOH profiles of subclones for HET (gray), LOH (green), NLOH (blue), or ASCNA (red). **j** CNV/LOH features shared by subclones of MG1 samples]

We used the TITAN program^4^ for intra-tumor subclone analysis. The germline heterozygous SNP loci (HET) across the genome were detected and the CNV and loss of heterozygosity (LOH) segments were jointy inferred from read depth and digital allele ratios at the HETs of the tumor WGS data. The new CNV and LOH types were reassessed at the multi-clone state that might be different from the previous single clone detection (Fig. 1d, Supplementary Fig. S2). As seen in Fig. 1e, the AAH4 and AD4 had ~30% normal cellular estimate based on the number of Het retained as normal sample. Each sample had two subclones. The subclone C2 of AAH4 shared variation features (copy number GAIN) with subclone C1 of AD4 (Fig. 1f). As seen in Fig. 1d, the AAH4 and AD4 had ~30% normal cellular estimate based on the number of Het retained as normal sample. Each sample had two subclones. The subclone C2 of AAH4 shared variation features (copy number GAIN) with subclone C1 of AD4 (Fig. 1f). Subclone C5 of AAH5 shared features with C5 of AD5 (red, copy number GAIN). Though we did not see a similar CNV pattern between AAH1 and AIS1 in CNV overview, the small C3 (0.28) of AAH1 shared features with the small C4 (0.26) of AIS1 in copy number GAIN. The SM-GGNs of patient 3 did not show co-occurring events in any subclone after the CNV/LOH deconstruction.

For verification, low-depth WGS DNA sequencing was performed on an additional 30 samples from 10 SM-GGN patients, two SM-GGNs and one paired normal controls of the lung tissues for each patient. The overview of somatic CNV shows a strong pattern in SM-GGN pairs of patients 1, 2, 4, and 10 (Fig. 1g). Interestingly, the LOH types showed more even pattern than did CNV types, as almost all SM-GGNs pairs shared ALOH except those in patient 10 (Fig. 1h). Two to five subclones were detected in the 20 GGNs samples. Patients 1, 2, 3, 5, 6, and 10 had more common features in the top 1 or 2 subclones compared to patients 7, 8, and 9. Patient 1 showed a parallel CNV pattern between MG1AAH and MG1AIS (Fig. 1i, j). This evidence supported a parallel lineage among the SM-GGNs of patients 1, 2, 3, 5, 6, and 10 and an independent lineage among the SM-GGNs of patients 7, 8, and 9.

Based on our data, we propose two models for the evolution of lung SM-GGNs. Supplementary Figure S3a depicts a model of independent lineage relationship in which two cancer-initiating cell clones (CIC) independently generate synchronous SM-GGNs. SM-GGNs can also arise in parallel from a single primary CIC clone (parallel lineage relationship) (Supplementary Fig. S3b). SM-GGNs arising via parallel lineage relationships may share high-impact variants (as these are more likely to be tissue-specific driver mutations), but their continued and separate evolution are expected to produce low-frequency, low-impact variants that would be unique to each lesion. The model also predicts that SM-GGNs with a parallel lineage relationship will have a high proportion of shared copy number aberrations and structural variants as well as a common subclone structure. Importantly, a shared subclone structure implies that many cells are from the same ancestor clone. Likewise, we acknowledge that various of analysis including RNA-seq, proteogenomics will be required for deeper research on SM-GGNs.

In summary, SM-GGNs in lung tumors present clinical challenges in surgery setting, therapy decision, and prognosis prediction.^5^ Comparing to our understanding of multiple metastasis tumors which were often found to share somatic driver mutations, we have much less knowledge about the molecular biology of how the multiple nodules occur. By deconstructing two types of genomic data (deep WGS:100X, and low-depth WGS:5X), our discoveries include shared CNV patterns and clonal structures between SM-GGNs. Our data support CNV correlation and cellular subclone estimation being an alternative and potentially more accurate and specific route for lineage determination.

## Supplementary information

SUPPLEMENTAL MATERIAL

## Data Availability

All the sequencing data have been deposited at the Sequence Read Archive (SRA, http://submit.ncbi.nlm.nih.gov/subs/sra/), which is hosted by the NCBI under the accession code SUB2009369.
