# Supplementary material for: Deconstructing the intra-tumor subclonal heterogeneity of lung synchronous ground-glass nodules using whole-genome sequencing
Source: Signal Transduct Target Ther. 2022 May 27;7:164. doi: 10.1038/s41392-022-00982-4 (PMC9135758; doi:10.1038/s41392-022-00982-4)
Supplement: Supplementary file 1 — SUPPLEMENTAL MATERIAL [file 41392_2022_982_MOESM1_ESM.docx]

Supplementary Materials for

Deconstructing the intra-tumor subclonal heterogeneity of lung synchronous ground-glass nodules using whole genome sequencing

Yijiu Ren1*, Minfang Song2*, Yunlang She1*, Huikang Xie2, Hui Zheng1, Chang Chen1^#^, Yiwen Zhang3^#^

Correspondence to: Yiwen Zhang, State Key Laboratory of Biotherapy, West China Hospital, Sichuan University and Collaborative Innovation Center for Biotherapy, Chengdu, 610041, China. Email: yiwenzhang@scu.edu.cn; Chang Chen, Department of Thoracic Surgery, Shanghai Pulmonary Hospital, Tongji University School of Medicine, Shanghai, 200443, China,

Email: changchenc@hotmail.com

**This PDF file includes:**

Materials and Methods

Figures. S1 to S3

Tables S1

Materials and Methods

Patients and tissue samples.

Frozen tissues for whole-genome sequencing were obtained from patients who underwent surgical resection at the Shanghai Pulmonary Hospital. Pathological diagnosis and staging were performed according to the 2015 International Association for the Study of Lung Cancer (IASLC)/American Thoracic Society (ATS)/European Respiratory Society (ERS) International Multidisciplinary Classification of Lung Adenocarcinoma and the TNM staging system of the IASLC, version 8^1^. Two pathologists reviewed all samples to confirm the histology and assess the tumor content. We selected only SM-GGNS patients with two GGN lesions, which were defined when all tumors exhibited GGN dominance with a consolidation/ tumor ratio <0.5 based on thin-section CT findings, with one diagnosed as AAH and another diagnosed as either AIS, MIA, or AD. The institutional Ethics Committee of the Shanghai Pulmonary Hospital approved the study (k16-264).

Whole-genome sequencing.

DNA was extracted using a QIAamp DNA Mini Kit (QIAGEN, Shanghai, China) and quantified with the Nanodrop system (Thermo Scientific, Shanghai, China). Matched normal lung tissue was used in each case as a control. Paired-end genome-wide sequencing (WGS) of the samples was performed at Novogene, Inc. (Beijing, China). A paired-end DNA library was generated using a Truseq Nano DNA HT Sample Preparation Kit (Illumina USA) following the manufacturer’s recommendations, and index codes were added to attribute sequences to each sample. Briefly, sonication of 1.0 μg tumor DNA samples was performed using a Covaris S220 sonicator (Massachusetts, USA) to generate fragments with an average size of 350 bp. Subsequently, the DNA fragments were end-polished, A-tailed, and ligated with the full-length adapter for Illumina sequencing, followed by further PCR (Polymerase Chain Reaction) amplification. After DNA were purified using SPRI beads from Agencourt, the libraries were analyzed for size distribution by an Agilent 2100 Bioanalyzer and quantified by real-time PCR (3 nM). Clustering of the index-coded samples was performed on a cBot Cluster Generation System using a Hiseq X HD PE Cluster Kit (Illumina) according to the manufacturer’s instructions. After cluster generation, the DNA libraries were sequenced on an Illumina Hiseq X platform, and 150 bp paired-end reads were generated. The target depth was 100× for deep-depth WGS, and 5× for all low-depth WGS.

Sequence quality check and mapping

All sequence reads were assessed for quality, including removing the adaptors, the reads that had more than 10% of “N”, and the read pair that had quality of less than 5 in more than 50% of bases. Valid sequencing data were mapped to the reference human genome (UCSC hg19) by Burrows-Wheeler Aligner (BWA) software^2^ to obtain the original mapping results stored in BAM format.

CNV and SNV analysis

Control-freec ^3^ was used to perform CNV detection. The somatic SNVs were detected by muTect,^4^ the somatic InDel were detected by Strelka,^5^For the low-depth WGS data, the HMMcopy ^6^ was used for copy number and segment estimate. The heat-maps of somatic CNVs were analyzed by GISTIC ^7^. Unsupervised hierarchical clustering of somatic CNVs was analyzed by Complex Heatmaps [https://github.com/-jokergoo/ComplexHeatmap] according to the study by Ni et al.^8^.

Cellular subclonal analysis

The cellular subclones were inferred using CNV and LOH joint analysis by TITAN program.^9^ TITAN is a statistical model for predicting segmental CNA and LOH from matched tumour and normal WGS data. The input to the model is the full set of germline heterozygous SNP loci (HET) and the corresponding read depth and allele ratios at these SNP positions from the tumour. The output is a set of segmental CNA and LOH, clonal cluster memberships, and estimated cellular prevalences. The tumour and sample cellular prevalences are defined as the proportion of the tumour cells and the proportion of the sample (including normal cells) that harbour a CNA/LOH event, respectively. We first identified the HET SNPs from the normal sample using samtools pileup. These HETs allows the detection of both CNA and LOH. The log Ratio of counts at loci of normal and tumor was used to infer the CAN and the allele ratio at loci of the tumor sample for LOH reference. Finally, a factorial hidden Markov model (HMM) searched the cellular subclones that harbor concurrent CNV/LOH and inferred the cellular prevalence.

References

1. Detterbeck, F.C., Franklin, W.A., Nicholson, A.G., Girard, N., Arenberg, D.A. *et al.* The IASLC Lung Cancer Staging Project: Background Data and Proposed Criteria to Distinguish Separate Primary Lung Cancers from Metastatic Foci in Patients with Two Lung Tumors in the Forthcoming Eighth Edition of the TNM Classification for Lung Cancer. *J Thorac Oncol* **11**, 651-665 (2016).

2. Li, H. & Durbin, R. Fast and accurate short read alignment with Burrows-Wheeler transform. *Bioinformatics* **25**, 1754-60 (2009).

3. Boeva, V., Popova, T., Bleakley, K., Chiche, P., Cappo, J. *et al.* Control-FREEC: a tool for assessing copy number and allelic content using next-generation sequencing data. *Bioinformatics* **28**, 423-5 (2012).

4. Cibulskis, K., Lawrence, M.S., Carter, S.L., Sivachenko, A., Jaffe, D. *et al.* Sensitive detection of somatic point mutations in impure and heterogeneous cancer samples. *Nat Biotechnol* **31**, 213-9 (2013).

5. Saunders, C.T., Wong, W.S., Swamy, S., Becq, J., Murray, L.J. *et al.* Strelka: accurate somatic small-variant calling from sequenced tumor-normal sample pairs. *Bioinformatics* **28**, 1811-7 (2012).

6. Ha, G., Roth, A., Lai, D., Bashashati, A., Ding, J. *et al.* Integrative analysis of genome-wide loss of heterozygosity and monoallelic expression at nucleotide resolution reveals disrupted pathways in triple-negative breast cancer. *Genome Res* **22**, 1995-2007 (2012).

7. Mermel, C.H., Schumacher, S.E., Hill, B., Meyerson, M.L., Beroukhim, R. *et al.* GISTIC2.0 facilitates sensitive and confident localization of the targets of focal somatic copy-number alteration in human cancers. *Genome Biol* **12**, R41 (2011).

8. Ni, X., Zhuo, M., Su, Z., Duan, J., Gao, Y. *et al.* Reproducible copy number variation patterns among single circulating tumor cells of lung cancer patients. *Proc Natl Acad Sci U S A* **110**, 21083-8 (2013).

9. Yin, X., Jing, Y., Cai, M., Ma, P., Zhang, Y. *et al.* Clonality, Heterogeneity, and Evolution of Synchronous Bilateral Ovarian Cancer. *Cancer Res.* **77**, 6551-6561 (2017).

Figure. S1. Imaging and pathology images of the five synchronous multiple ground-glass nodules for the whole-genome sequencing. The left side shows the location of all lesions in the lung models, with colorful dots representing the lesions. The middle side shows the CT scans of all lesions, with the yellow block chart showing the exact locations of the lesions. The right side shows the pathology sections of all lesions. P represents each patient.


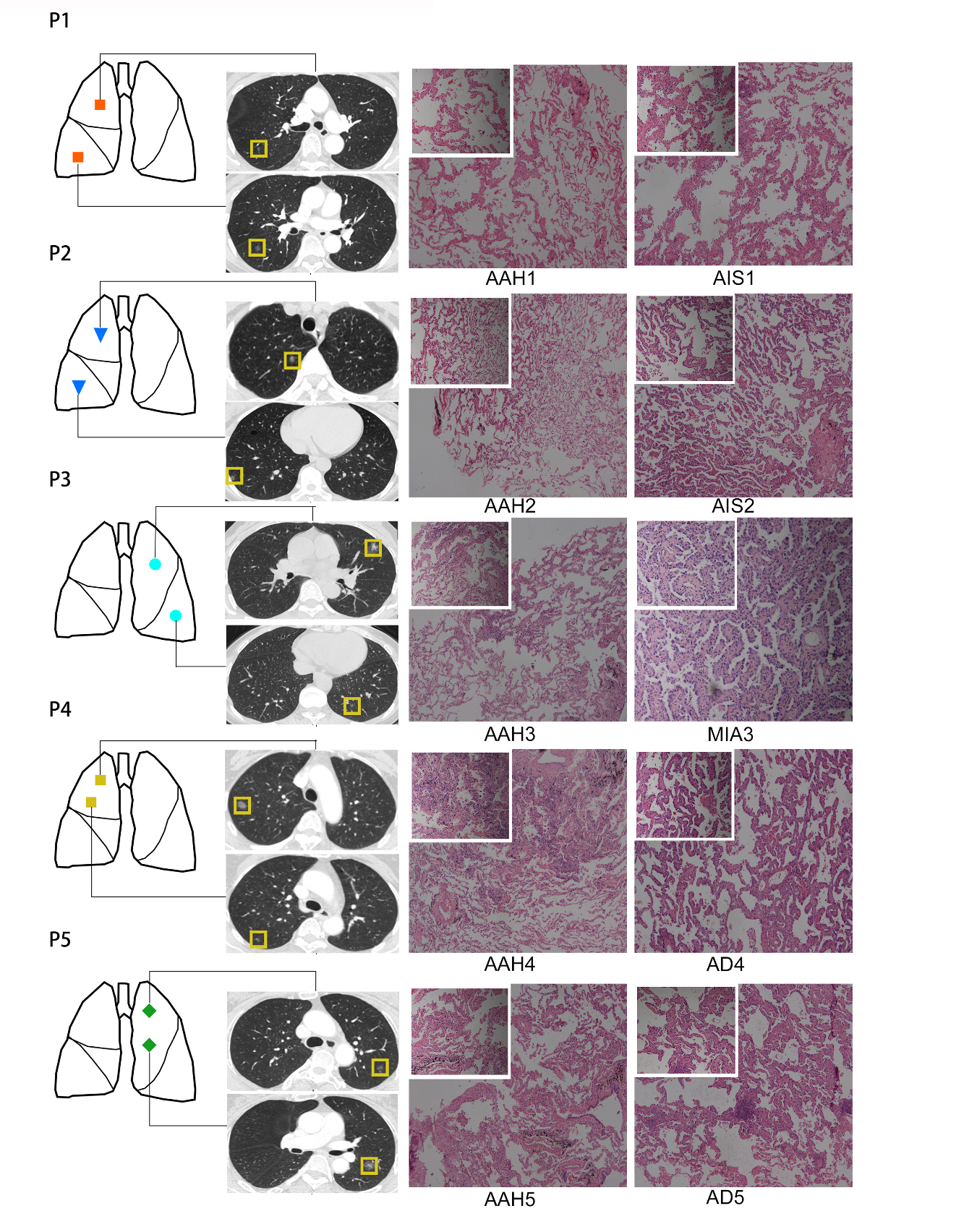


Figure. S2. Clonal structure of all patients. a AAH1 (left) and AIS1 (right) of patient 1. b AAH2 (left) and AIS2 (right) of patient 2. c AAH3 (left) and MIA3 (right) of patient 3. d AAH4 (left) and AD4 (right) of patient 4. e AAH5 (left) and AD5 (right) of patient 5. Top panel: Copy-number is represented as the log ratio of tumor and normal read depth. Discrete copy-number status shown is predicted as either a hemizygous deletion (HEMD; green), copy neutral (NEUT; blue), or gain/amplification (AMP; red). Middle panel: Allelic ratios are computed as the proportion of reads matching the reference genome. The LOH status shown is HET (grey), LOH (green), NLOH (blue), or ASCNA (red). Lower panel: the sample cellular prevalence estimates (proportion of sample) for a subclonal cluster ‘Z1’ and ‘Z2”.


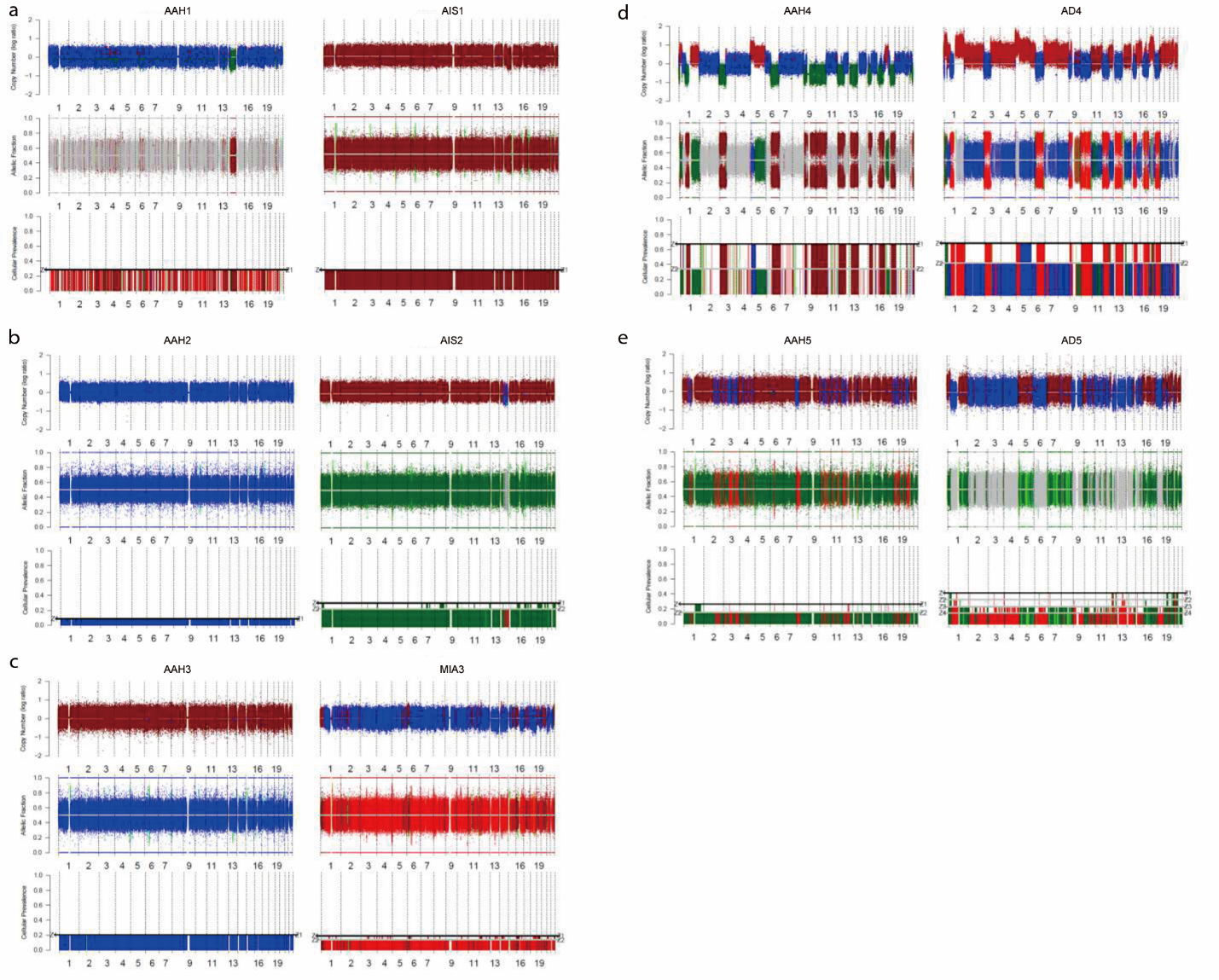


Figure. S3. Parallel and independent lineage models. a In independent lineage, the synchronous multiple GGNs originated from different cancer stem cells. Their cellular subclones and the somatic genomics that initiated the tumor had less in common. b In parallel lineage, though a large number of new genomic alterations occurred as the ancestor cell evolved, a portion of genomic features was retained in subclones (for example, the subclone with the same color). The genomic features that originally drove (high impact) the cancer ancestor will descend into the subclones. In both cases, the multiple GGNs will share the same individual germline variations.


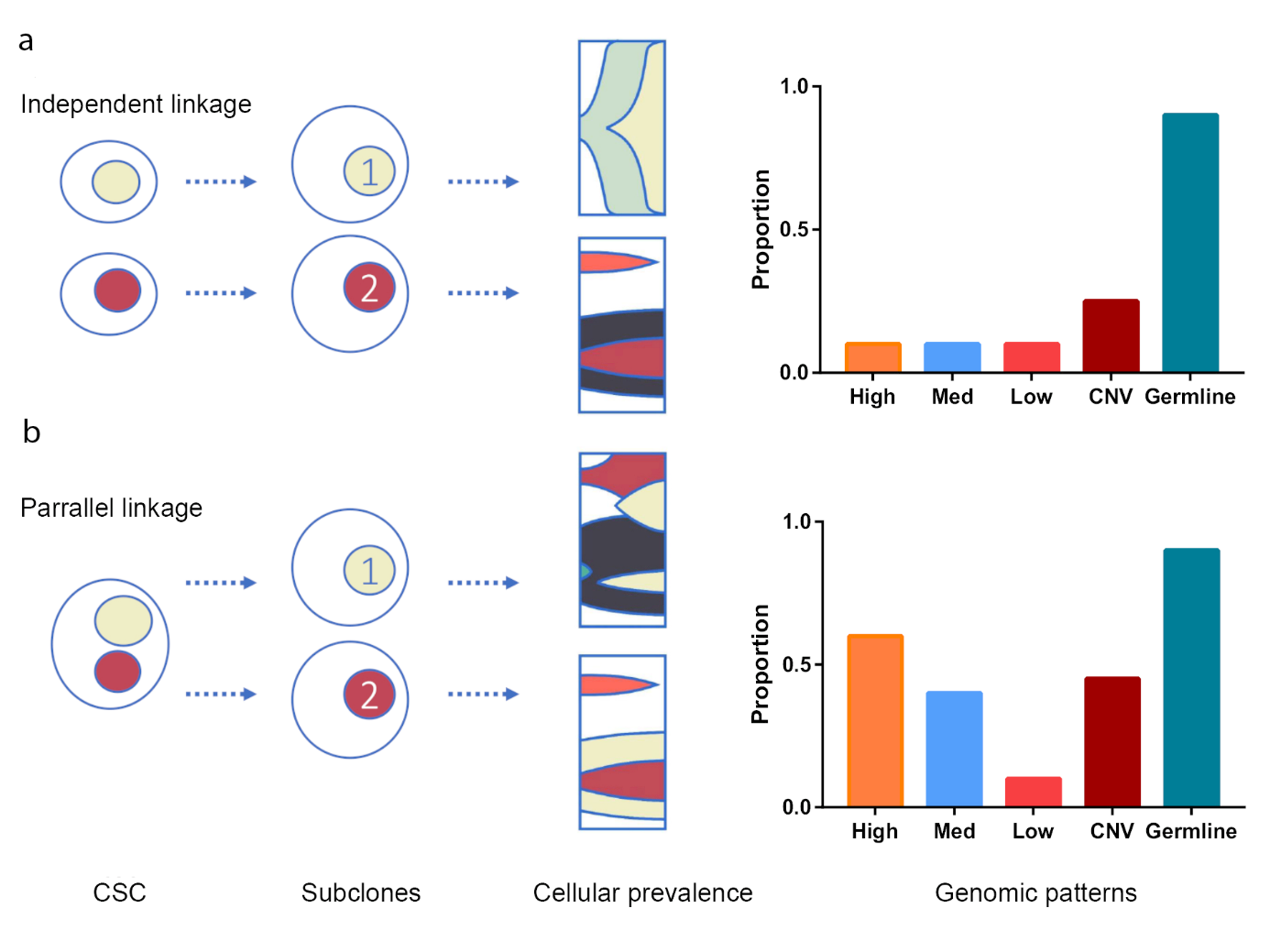


Table S1.

**Table S1. Patient clinical information**

| **Patient** | **corhort** | **GGN** | **N** | **T** | **cm** | **sample** | **Normal** | **Age** | **Gender** | **smoking** | **month** | **DF** |
| --- | --- | --- | --- | --- | --- | --- | --- | --- | --- | --- | --- | --- |
| P1 | deep_WGS | AAH | N0 | T1 | 0.50 | frozen | lung | 68 | Female | None | 29 | 0 |
| P1 | deep_WGS | AIS | N0 | T1 | 0.80 | frozen | lung | 68 | Female | None | 29 | 0 |
| P2 | deep_WGS | AAH | N0 | T1 | 0.40 | frozen | lung | 49 | Female | None | 30 | 0 |
| P2 | deep_WGS | AIS | N0 | T1 | 0.80 | frozen | lung | 49 | Female | None | 30 | 0 |
| P3 | deep_WGS | AAH | N0 | T1 | 0.50 | frozen | lung | 59 | Female | None | 29 | 0 |
| P3 | deep_WGS | MIA | N0 | T1 | 0.90 | frozen | lung | 59 | Female | None | 29 | 0 |
| P4 | deep_WGS | AAH | N0 | T1 | 0.50 | frozen | lung | 56 | Female | None | 29 | 0 |
| P4 | deep_WGS | AD | N0 | T1 | 1.30 | frozen | lung | 56 | Female | None | 29 | 0 |
| P5 | deep_WGS | AAH | N0 | T1 | 0.50 | frozen | lung | 59 | Female | None | 27 | 0 |
| P5 | deep_WGS | AD | N0 | T1 | 1.6 | frozen | lung | 59 | Female | None | 27 | 0 |
| MG1 | low_WGS | AAH | N0 | T1 | 0.50 | frozen | lung | 56 | Female | None | 24 | 0 |
| MG1 | low_WGS | AIS | N0 | T1 | 0.80 | frozen | lung | 56 | Female | None | 24 | 0 |
| MG2 | low_WGS | AAH | N0 | T1 | 0.50 | frozen | lung | 61 | Male | None | 24 | 0 |
| MG2 | low_WGS | AIS | N0 | T1 | 0.90 | frozen | lung | 61 | Male | None | 24 | 0 |
| MG3 | low_WGS | AIS | N0 | T1 | 1.00 | frozen | lung | 59 | Male | None | 24 | 0 |
| MG3 | low_WGS | AAH | N0 | T1 | 0.40 | frozen | lung | 59 | Male | None | 24 | 0 |
| MG4 | low_WGS | AIS | N0 | T1 | 0.70 | frozen | lung | 57 | Female | None | 24 | 0 |
| MG4 | low_WGS | AAH | N0 | T1 | 0.50 | frozen | lung | 57 | Female | None | 24 | 0 |
| MG5 | low_WGS | AAH | N0 | T1 | 0.50 | frozen | lung | 63 | Female | None | 25 | 0 |
| MG5 | low_WGS | AD | N0 | T1 | 1.50 | frozen | lung | 63 | Female | None | 25 | 0 |
| MG6 | low_WGS | AAH | N0 | T1 | 0.50 | frozen | lung | 65 | Male | None | 25 | 0 |
| MG6 | low_WGS | AIS | N0 | T1 | 0.60 | frozen | lung | 65 | Male | None | 25 | 0 |
| MG7 | low_WGS | MIA | N0 | T1 | 1.10 | frozen | lung | 66 | Female | None | 26 | 0 |
| MG7 | low_WGS | AAH | N0 | T1 | 0.50 | frozen | lung | 66 | Female | None | 26 | 0 |
| MG8 | low_WGS | AAH | N0 | T1 | 0.50 | frozen | lung | 54 | Female | None | 26 | 0 |
| MG8 | low_WGS | AD | N0 | T1 | 1.50 | frozen | lung | 54 | Female | None | 26 | 0 |
| MG9 | low_WGS | AIS | N0 | T1 | 0.80 | frozen | lung | 56 | Female | None | 26 | 0 |
| MG9 | low_WGS | AAH | N0 | T1 | 0.50 | frozen | lung | 56 | Female | None | 26 | 0 |
| MG10 | low_WGS | AAH | N0 | T1 | 0.50 | frozen | lung | 59 | Female | None | 27 | 0 |
| MG10 | low_WGS | MIA | N0 | T1 | 1.00 | frozen | lung | 59 | Female | None | 27 | 0 |

*N: N-stage; T: T-stage; Month: follow up time; DF: disease free.
